# Foliar Application of NH_4_^+^/NO_3_^–^ Ratios Enhance the Lodging Resistance of Soybean Stem by Regulating the Physiological and Biochemical Mechanisms Under Shade Conditions

**DOI:** 10.3389/fpls.2022.906537

**Published:** 2022-07-22

**Authors:** Ali Raza, Chunying Yin, Muhammad Ahsan Asghar, Muhammad Ihtisham, Iram Shafiq, Bin Cheng, Abuzar Ghafoor, Hafiz Hassan Javed, Tauseef Iqbal, Nawab Khan, Weiguo Liu, Wenyu Yang

**Affiliations:** ^1^Key Laboratory of Crop Ecophysiology and Farming System in Southwest China, Ministry of Agriculture, Sichuan Agricultural University, Chengdu, China; ^2^Sichuan Engineering Research Center for Crop Strip Intercropping System, Chengdu, China; ^3^CAS Key Laboratory of Mountain Ecological Restoration and Bioresource Utilization and Ecological Restoration and Biodiversity Conservation Key Laboratory of Sichuan Province, Chengdu Institute of Biology, Chinese Academy of Sciences, Chengdu, China; ^4^Chengdu Institute of Biology, University of Chinese Academy of Sciences, Beijing, China; ^5^Department of Biological Resources, Agricultural Institute, Centre for Agricultural Research, ELKH, Martonvásár, Hungary; ^6^College of Landscape Architecture, Sichuan Agricultural University, Chengdu, China; ^7^Chengdu Da Mei Seeds Co., Ltd., Chengdu, China; ^8^College of Agriculture and Biotechnology, China Agricultural University, Beijing, China; ^9^College of Agronomy, Sichuan Agricultural University, Chengdu, China; ^10^College of Management, Sichuan Agricultural University, Chengdu, China

**Keywords:** soybean, shade stress, NH_4_^+^/NO_3_^–^, lignin enzymes, stem lodging resistance

## Abstract

Shading is one of the most chronic restrains which can lead to the lodging of intercropped plants. In order to increase the soybean stem lodging resistance, a 2-year field trial was conducted to evaluate the impact of different ratios and concentrations of NH_4_^+^/NO_3_^–^ on the morpho-physiological and biochemical characteristics of soybean stem under shade conditions. The total 5 ratios of NH_4_^+^/NO_3_^–^ were applied as follows: T0 = 0/0 (control), T1 = 0/100 (higher ratio), T2 = 25/75 (optimum), T3 = 50/50 (optimum), and T4 = 75/25 (higher ratio) as a nitrogen source. Our findings displayed that the T2 (25/75) and T3 (50/50) treatments alleviated the shading stress by improving the photosynthetic activity, biomass accumulation, carbohydrates contents, and lignin related enzymes (POD, CAD, and 4Cl) which led to improvement in stem lodging resistance. The correlation analysis (*p* ≤ 0.05, *p* ≤ 0.01) revealed the strong relationship between lodging resistance index and stem diameter, stem strength, lignin content, photosynthesis, and lignin related enzymes (POD, CAD, and 4CL) evidencing the strong contribution of lignin and its related enzymes in the improvement of lodging resistance of soybean stem under shade conditions. Collectively, we concluded that optimum NH_4_^+^/NO_3_^–^ ratios (T2 and T3) can boost up the lodging resistance of soybean stem under shade stress.

## Introduction

Shading is one of the common constrains which can cause the lodging of soybean intercropped with maize plants (Raza et al., 2020). The microclimate of soybean is affected due to the high canopy of maize plants, which hampers the light penetration into the soybean canopy in comparison to normal light (Yang et al., 2014). The low light availability to the soybean canopy hinders its growth and competition for nutrient uptake at the vegetative stage (Hussain et al., 2019a; Liu et al., 2019). Shade is a persistent abiotic stress which decreases the crop yield and productivity in intercropping systems (Yang et al., 2018; Hussain et al., 2020). Soybean yield is affected by up to 87% due to shade stress in an intercropping system as compared to mono-cropping (Yang et al., 2017). Under shade conditions, weaker stem strength leads to a higher lodging stress in soybean plants (Wen et al., 2020). Lodging is also one of the most chronic constraints in intercropping systems which hinders soybean growth at the early stage (Liu et al., 2018). Previous studies explained that stem lodging significantly hampered the process of photosynthesis at the grain filling stage (Weng et al., 2017). It has been observed that the length of soybean stems is increased up to 45.75% under shade conditions which caused lodging and eventually decreased the final yield by 20–40% (Liu et al., 2017b). Stem lodging decreases the production of grain and increases the cost of harvest and deteriorates the grain quality (Berry and Spink, 2012). Another study described that lodging can reduce the grain production by up to 80% of the total crop yield (Acreche and Slafer, 2011). The photosynthetic rate was also affected under shade stress due to blockage of electron transportation from photosystem II to photosystem I, and consequently the rate of electron transport, rubisco activity, and ATP production also decreases (Yao et al., 2017a; Huang et al., 2018). Shade stress reduces the non-structural carbohydrates content such as sucrose, soluble sugars, and starch which help in energy and structural carbohydrates accumulation (Wu et al., 2017). Our previous studies verified that structural carbohydrates (cellulose), lignin polymer, and non-structural carbohydrates (soluble sugars and sucrose) are the main components of stem strength (Liu et al., 2016; Hussain et al., 2020). Shade stress had a significant effect on lignin metabolism and biosynthesis through modifying the activities of lignin related enzymes, i.e., peroxidase (POD), 4-coumarate CoA ligase (4CL), cinnamyl alcohol dehydrogenase (CAD), and phenylalanine ammonia-lyase (PAL) (Koyama et al., 2012; Liu et al., 2017b).

Nitrogen is a key macronutrient that is necessary for all plant growth and development. The fundamental sources of N (nitrogen) are NH_4_^+^ (ammonium) and NO_3_^–^ (nitrate) which prompt the plant growth (Chen et al., 2011, 2018). Nitrate (NO_3_^–^) promotes plant growth better than ammonia (NH_4_^+^), however, if both are supplied collectively then plants grow even better (Raza et al., 2021). The optimum concentration of NH_4_^+^ can also be influenced by abiotic factors such as light intensity, pH of the soil or nutrient solution (Urlić et al., 2017). The uptake of different forms of N occurs through specific metabolic pathways which integrate in different ways with the carbon metabolism and alter the plant tissue composition (Krapp, 2015). Under shade conditions because of low light interception, the reduction activities of NO_3_^–^ may decelerate and the NO_3_^–^ content increases, which results in a scarcity of reducing bodies and carbon skeletons (Demšar et al., 2004). The NH_4_^+^ and NO_3_^–^ increase the plant resilience to multiple environmental stresses including salinity, drought, alkalinity, and low light stress and also alleviate the higher concentration of CO_2_ (Esteban et al., 2016; Raza et al., 2021). There are a number of strategies which have been reported recently to overcome the lodging stress of soybean including proper sowing time and planting density (Ahmed et al., 2020) and use of chemicals to reduce the plant height (Xu et al., 2017). As nitrogen is a key macronutrient for plant growth, the optimization of nitrogen fertilizers such as NH_4_^+^/NO_3_^–^ might be useful for increasing the lodging resistance of soybean under shade conditions. Therefore, considering the beneficial effects of NH_4_^+^/NO_3_^–^ as a nitrogen source for plants, we hypothesized that foliar application of NH_4_^+^/NO_3_^–^ can enhance the soybean lodging resistance by regulating the morpho-physiological and biochemical attributes under shade conditions. The objectives of this research were to evaluate the lodging resistance of soybean stem and variations in the photosynthetic efficiency, carbohydrates content, stem strength, lignin, and cellulose content, and the activities of lignin related enzymes in soybean, grown under normal light and shade conditions with application of different foliar ratios of NH_4_^+^/NO_3_^–^.

## Materials and Methods

**FIGURE 1:**
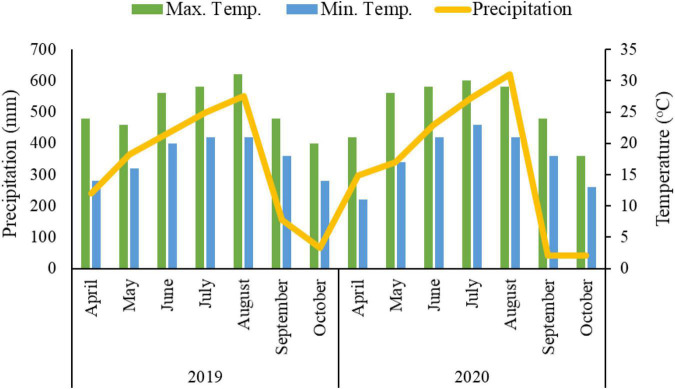
Climatic data showing mean monthly precipitation (mm), maximum and minimum temperature (°C) of growing seasons 2019–2020.

A 2-year field trial was conducted in 2019–2020 at the Chongzhou research station of Sichuan Agricultural University (103°39′E, 30°33′N). The climatic conditions of the research station were subtropical and humid. The annual average temperature, sunshine, and annual mean rainfall were 15.9°C, 1,161.5 h, and 1,012.4 mm, respectively. The climatic data of both growing seasons (2019–2020) is presented (Figure 1). The soil was classified as purple and clayey soil at the experimental site, with total nitrogen content 1.6 g kg^–1^, total phosphorus content 1.3 g kg^–1^, total potassium 15.2 g kg^–1^, available nitrogen 299.5 mg kg^–1^, available phosphorus 1.3 g kg^–1^, available potassium 169.4 mg kg^–1^, content of organic matter 24.3 g kg^–1^, and a pH of 7.1.

### Experimental Description

**FIGURE 2:**
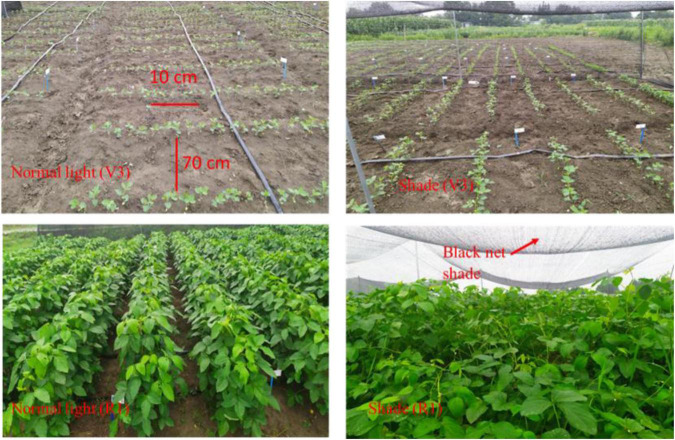
The experiment layout of soybean plantation was shown at V3 stage (fully expanded trifoliate) and R1 stage (flowering stage) under normal light and shade conditions.

Soybean cultivar (Nan-99-6) obtained by Sichuan Agricultural University was grown in the field under a complete randomized block design (RCBD) with three replications. Soybean crop was grown in two planting conditions (normal light and shade conditions) as a main factor (Figure 2), and a total of five treatments divided into control, optimum, and higher ratios on the basis of concentration [T0 = 0/0 (control), T1 = 0/100 (higher ratio), T2 = 25/75 (optimum), T3 = 50/50 (optimum), and T4 = 75/25 (higher ratio)] of different ratios of NH_4_^+^/NO_3_^–^ as a secondary factor. The plant-to-plant distance was 10 cm and the row-to-row distance was kept at 70 cm. Every treatment included three strips of soybean and the size of one strip was 7 m × 2 m with a total plot size of 7 m × 6 m (42 m^2^) for both light and shade conditions. Foliar application of different ratios of NH_4_^+^/NO_3_^–^ (T0 = 0/0, T1 = 0/100, T2 = 25/75, T3 = 50/50, and T4 = 75/25) with 5 mM total concentration of N being sprayed to soybean at V3 (fully expanded trifoliate), V5 (five trifoliate vegetative stage), and R1 (flowering stage) stages. The different NH_4_^+^/NO_3_^–^ ratios treatments contained a total nitrogen concentration of NH_4_^+^ and NO_3_^–^ in the form of NH_4_(SO_4_)_2_ and KNO_3_, respectively. The following were treatment details used for this experiment: T0 = control; T1 = 5 mM NO_3_^–^; T2 = 1.25 mM NH_4_^+^/3.75 mM NO_3_^–^; T3 = 2.5 mM NH_4_^+^/2.5 mM NO_3_^–^; T4 = 3.75 mM NH_4_^+^/1.25 mM NO_3_^–^, both under both light conditions. The first foliar spray of treatments was applied when the first trifoliate leaves appeared and treatments were sprayed at the intervals of 10–12 days, for a total of three times. After 1 day of germination, soybean seedlings were subjected to the shade condition, which was provided by one layer of black net shade. During the experiment, the normal light was at 1,250 μmol/m^2^/s and for shaded plants the light intensity was at 350 μmol/m^2^/s. The light intensity was measured by HR350 (Hipoint Inc., Gaoxiong, Taiwan) (Wen et al., 2020). After 40 days, at V5 (vegetative stage), shade was removed and after shade removal foliar treatments were sprayed to soybean plants to check how soybean plants responded to treatments. The sampling was done at V5 (vegetative stage) to investigate the impact of growth stages on the experimental variables. The normal light condition was provided by full sunlight.

### Sampling and Measurements

#### Biomass Accumulation, Stem Diameter, and Plant Height

Ten uniform soybean plants were taken from each plot at the V5 stage (five trifoliate vegetative stage) to measure the morphological parameters. The fresh biomass of plants was weighed, and samples were kept in an oven at a temperature of 105°C for 15 min and then placed at 80°C to obtain a constant value of weight. The stem diameter of soybean plants was measured at the center point of the second internode above the belowground with a Vernier caliper. The height of soybean plants was measured with a metric ruler.

#### Pigment Analysis and Relative Chlorophyll Content (SPAD Value)

Soybean fully expanded leaves were collected at the V5 stage (five trifoliate vegetative stage) for chlorophyll pigments (Chlorophyll a), Chlorophyll b, and carotenoids) and pigments were extracted with 80% acetone and measured at 663, 645, and 470 nm wavelengths in a spectrophotometer (UV-2250; Kyoto, Japan) (Raza et al., 2021). Relative chlorophyll content (SPAD value) was recorded by using a chlorophyll meter, SPAD 502 (Minolta, Japan).

#### Photosynthetic Parameters and Fluorescence Characteristics

The photosynthetic parameters, such as photosynthesis rate (*Pn*), stomatal conductance (*gs*), transpiration rate (*E*), and intercellular CO_2_ concentration (*C*_*i*_) were measured at the V5 stage (five trifoliate vegetative stage) with a moveable photosynthetic system (LI-6400; LI-COR, Lincoln, NE, United States). The readings were taken from 9:00 to 11:00 a.m. on a sunny day. The LI-COR was calibrated at the flow rate of 500 μmol mol^–1^, PAR = 1,000 μmol m^–2^s^–1^, leaf temperature = 26°C, and ambient CO_2_ concentration = 400 μmol mol^–1^.

A software Flour Technologia (Fluor Images, United Kingdom; version 2.2.2.2) was used to measure the chlorophyll fluorescence. Fully expanded soybean leaves were taken and wrapped in a foil of aluminium and kept in an icebox to stop light penetration. Leaves were placed in the measuring box and flour image software was used to measure the fluorescence characteristics; maximum quantum yield in the dark (Fv/Fm), PS II (photosystem II), photochemical quenching (qP), and electron transport rate (ETR). The maximum fluorescence (Fm) was recorded at a 10 Hz saturation blue pulse, and the light adaption time was 10–15 min. The actinic light (1,000 mmol m^–2^s^–1^) was kept constant for all the samples (Hussain et al., 2019a).

#### Carbohydrates Content

The fresh soybean leaves were taken at the V5 stage (five trifoliate vegetative stage) and powdered in liquid nitrogen with a pestle and mortar to measure the carbohydrate content (starch, reducing sugars, and sucrose). After grinding the samples, 100 mg of each sample was taken and 6 mL of 80% ethanol was added and kept in a water bath for 40 min at 80°C. When it boiled, it was then cooled-down and centrifuged for 5 min at 5,000 rpm. The supernatant was taken out in 50 ml test tubes and made up the solution up to 50 ml of 80% ethanol. A charcoal solution was used for de-colorization, and samples were used to measure the starch, sucrose, and reducing sugars using the previously published method (Asghar et al., 2020).

#### Lodging Resistance Index and Stem Snapping Resistance

The lodging resistance index was recorded by using the following formula, lodging resistance index = snapping resistance ÷ (main stem length × aboveground fresh weight). For snapping resistance, ten uniform soybean plants were taken from each treatment. The stem snapping resistance was recorded from the 3rd internode to the 5th internode with a digital stem force tester (YYD-1, Zhejiang Top Instrument Hangzhou, China).

#### Measurement of C/N Ratio

Dried stem samples were milled (80 mg) and used to measure C and N ratios with a nitrogen and carbon analyzer (NC vario MACRO cube, ELEMENTAR, Germany).

#### Lignin Content

A kit method (Lignin Content Test Kit, Qi Yi, Shanghai, China) was used for the determination of lignin content with some changes. The dried stem powder (2 mg) was weighed into a centrifuge tube of 2.5 ml and 20 μl perchloric acid and 500 μl sulfuric acid were mixed. Then, the tube was closed with sealing film, mixed completely, and after every 10 mins, shaken in a water bath for 40 mins at 80°C and cooled-down. Afterward, 0.5 ml of every sample was shifted into a 50 ml centrifuge tube and a 995 μl solution of NaOH was added. The 200 μl supernatant was transferred into a 96 well plate for lignin determination. The value of absorbance for lignin content was recorded at 280 nm by the ELISA (SpectraMaxi3x, China). The value of lignin content was measured according to the following formula:


Lignin⁢Content⁢(mg/g)= 0.075×(Δ⁢A-0.0068)⁢/⁢W×T

ΔA, light absorbance value; W, weight of dry sample; T, dilution ratio.

#### Cellulose Content

For determination of cellulose content, dried soybean stems were grounded and weighed at about 300 mg and moved into a 2.5 ml tube. Initially, the sample was homogenized at room temperature by adding 1 ml of 80% ethanol and the reaction was started for 20 mins at 95°C. Then, the samples were cooled down, centrifuged at 4,000 rpm for 10 mins at 25°C, and the supernatants were disposed of. After that, 1.5 ml of 80% ethanol and acetone were added to wash out the precipitation, and then vortexed for about 2 min and the supernatant was discarded through centrifugation at 4,000 rpm for 10 mins at 25°C. Then, the starch was removed by soaking the precipitate into 1 ml dimethyl sulfoxide solution for 15 h and was again centrifuged at 4,000 rpm for 10 min at 25°C. Afterward, the cell wall material (CWM) was dried and weighed up to about 5 mg. To homogenize and shift the homogenate into the tube, 0.5 ml of distilled water was added to the tube. Afterward, concentrated sulfuric acid at about 0.75 ml was added and kept for 30 mins, and centrifuged at 4°C and 8,000 rpm for 10 min. The samples were diluted after centrifugation with 70 μl of anthrone ethyl acetate, and 630 μl of concentrated sulfuric acid added, and placed in a water bath for 10 min at 95°C (sealed firmly to inhibit water loss). At the end, it was cooled down at room temperature and the value of absorbance was recorded at 620 nm. The cellulose content value was measured by the following formula.


Cellulose⁢content⁢(mg/g)= 4.76⁢(Δ⁢A+ 0.0043)/W

ΔA, value of light absorbance; W, weight of dry sample.

#### Determination of Lignin Related Enzymes

For the determination of lignin related enzymes activity, fresh samples of stem from the 3rd to 5th internodes were collected and kept in the refrigerator at −80°C. An ELISA test kit (GeRuwasi, Shuzhou) was used to check the lignin enzymes (POD, CAD, and 4Cl) activity (Liu et al., 2019).

#### Grain Yield

Soybean grain yield was measured at the maturity stage, 10 plants were harvested from every plot and the number of pods, 100-seed weight, and seed yield (g/m^2^) were recorded. An electric weighing balance was used to measure the seed weight and total yield g/m^2^.

### Statistical Analysis

All the data documented were subjected to analysis of variance was performed using SPSS software (version 20.0 Chicago, IL, United States). The graphical presentation was done using Microsoft Excel 2016 software. The least significant difference (LSD) to compare all the means among different variables was presented at a probability level of *p ≤ 0.05. Pearson’s correlation coefficient was used to determine the relationship between the stem lodging resistance and morpho-physiological and biochemical activities of soybean with a significance level of *p* ≤ 0.05, *p* ≤ 0.01.

## Results

### Total Biomass Accumulation

**FIGURE 3:**
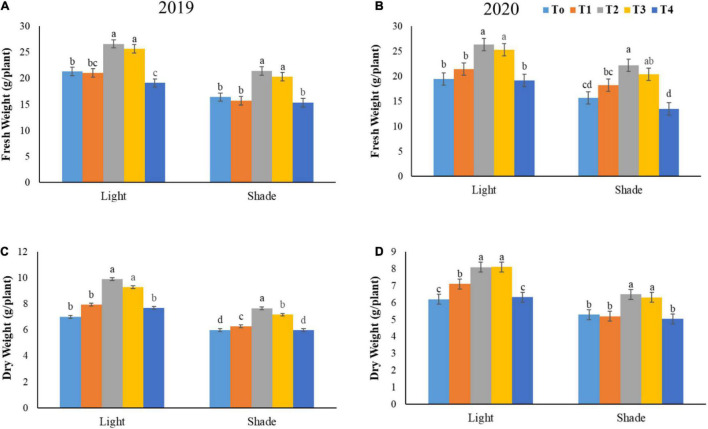
Effect of various NH_4_^+^/NO_3_^–^ ratios on fresh weight **(A,B)**, and dry weight **(C,D)** under light and shade. Different treatment levels T0, T1, T2, T3, and T4 characterize the ratios of NH_4_^+^/NO_3_^–^ 0/0, 0/100, 25/75, 50/50, and 75/25, respectively. All of the results are the mean of three replications. The bars represent the ± SD (standard deviation). A significant difference (*p < 0.05) between treatments is shown by different lowercase letters above bars.

Our findings demonstrated that foliar application of different ratios of NH_4_^+^/NO_3_^–^ significantly increased the biomass of soybean plants under both light regimes (Figure 3). The fresh biomass accumulation significantly accelerated at T2 by 38.47% and 30.10% as compared to T4 and T0 under normal light, respectively, in both years 2019–2020 cumulatively. Under shade conditions the value of fresh biomass was also enhanced at T2 by 51.07 and 21.71% as compared to T4 and T0, respectively, in both years.

Furthermore, dry biomass accumulation also increased at T2 level as compared to T0, T1, and T4 levels under both light environments. In both the studied years (2019–2020), the value of dry biomass prompted by 19.56, 36.21, and 28.33% at T2 in relation to T1, T0, and T4 under light condition, respectively. On the other hand, under shade condition dry biomass positively enhanced at T2 and T3 by 28.31 and 21.24% as compared to T4 in both years. More importantly, our results depicted those optimum ratios (T2 = 25/75 and T3 = 50/50) of NH_4_^+^/NO_3_^–^ had a significant effect on the total biomass accumulation as compared to higher NH_4_^+^/NO_3_^–^ T1 and T4 (T1 = 0/100 and T4 = 75/25) ratios in both years, under normal and shade conditions.

### Plant Height and Stem Diameter

**FIGURE 4:**
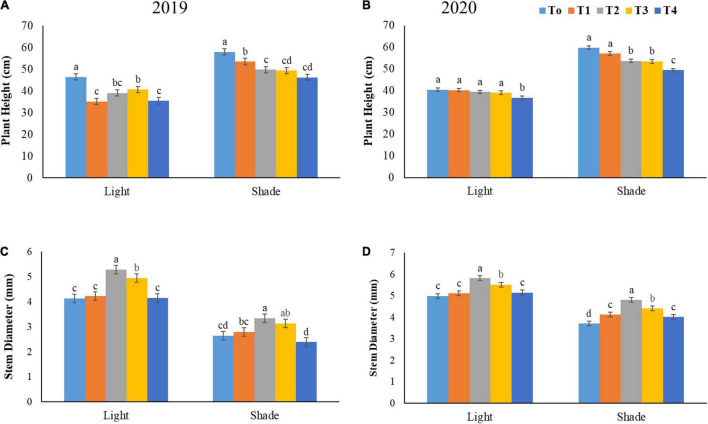
Impact of different NH_4_^+^/NO_3_^–^ ratios on plant height **(A,B)**, and stem diameter **(C,D)** under light and shade. Different treatment levels T0, T1, T2, T3, and T4 characterize the ratios of NH_4_^+^/NO_3_^–^ 0/0, 0/100, 25/75, 50/50, and 75/25, respectively. All of the results are the mean of three replications. The bars represent the ± SD (standard deviation). A significant difference (*p < 0.05) between treatments is shown by different lowercase letters above bars.

Increment in the stem height is a typical shade adaptive response of soybean plants under low light condition. In this experimentation, the plant height increased significantly under shade conditions by 35.00% relative to normal light conditions (Figure 4A). Interestingly, our results showed that foliar application of different ratios of NH_4_^+^/NO_3_^–^ significantly reduced the plant height under shade conditions when compared with control treatment. Optimum ratios of treatments (T2 and T3) significantly inhibited the plant height in both years by 10.82 and 8.98% as compared to control under normal light conditions. Furthermore, under shade conditions, the plant height was significantly reduced at T2 by 13.72 and 6.90% as compared to T0 and T1.

Our study revealed that, under shade conditions, the stem diameter was significantly reduced by 43.55% when compared to normal light at the average value of T0 (control) treatment for both years (2019–2020). However, foliar spray of different NH_4_^+^/NO_3_^–^ ratios improved the stem diameter of soybean in both light conditions (Figure 4B). In both studied years, a significant increment in stem diameter was observed at T2 by 21.71 and 19.35% as compared to T0 and T4, under normal light conditions as showed in Figures 4C,D. However, the stem diameter decreased at T4 and T0 by 12.47 and 14.69% in relation to T3 under normal light condition. Furthermore, under shade conditions the stem diameter increased at T2 by 22.89% as compared to higher ratio (T4) in both years.

### Total Carbohydrates Content, Lignin, Cellulose, and C/N Ratio

The impact of different ratios of NH_4_^+^/NO_3_^–^ on total carbohydrates content including sucrose, reducing sugars, and starch was studied under two light levels (normal light and shade condition). A significant difference was seen between different treatments, the content of C/N ratios, and carbohydrate content (Table 1). In both years, the sucrose and starch contents increased by 41.59 and 46.62%, and 23.94 and 21.02% at T2 treatment relative to T0 and T4, respectively, under normal light conditions. Contrarily, the reducing sugars content decreased at T1 and T4 level by 16.72 and 28.22% over T2 under normal light conditions in 2019–2020, respectively. Under shade conditions, the higher ratios of NH_4_^+^/NO_3_^–^ (T1 and T4) resulted in lower total carbohydrates (starch, sucrose, and reducing sugars) contents in comparison to optimum ratios (T2 and T3). More specifically, the increment in starch and reducing sugars content was observed by 17.60 and 16.60% in T2 and T3 treatment relative to T4 in both growing seasons (2019–2020), under shade conditions. Furthermore, the sucrose content prompted by 46.10 and 38.03% at T2 relative to T1 and T0, respectively, under shade conditions for both years (2019–2020) cumulatively.

**TABLE 1:** Effect of varied NH_4_^+^/NO_3_^–^ ratios on total carbohydrates and C/N ratios under different light conditions.

	Treatments (T)		Sucrose (mg/g)	Starch (mg/g)	Reducing sugars (mg/g)	C/N ratio
2019	Normal light	T0	5.93 c	29.38 c	23.67 c	1.57 c
		T1	7.13 b	32.89 b	24.40 b	1.82 b
		T2	8.65 a	37.95 a	27.79 a	1.88 b
		T3	8.43 a	36.7 a	27.27 a	1.98 a
		T4	5.61 c	30.61 bc	23.72 bc	1.81 b
		Average	7.15	33.50	25.37	1.79
	Shade condition	T0	8.53 c	41.02 bc	20.59 c	1.58 bc
		T1	6.63 bc	42.39 b	22.71 c	1.53 bc
		T2	8.97 a	45.48 a	25.34 a	1.89 a
		T3	8.42 ab	45.06 a	25.16 a	1.62 b
		T4	5.92 c	39.24 c	20.99 bc	1.47 c
		Average	7.69	42.63	22.95	1.61
2020	Normal light	T0	6.33 b	29.57 d	23.53 c	1.18 cd
		T1	7.19 ab	32.55 c	24.49 b	1.25 bc
		T2	8.71 a	38.55 a	27.69 a	1.61 a
		T3	8.43 a	36.29 b	27.10 b	1.51 ab
		T4	6.23 b	28.94 d	22.73 c	1.19 c
		Average	7.37	33.18	25.10	1.68
	Shade condition	T0	4.52 b	23.79 b	19.79 b	1.20 b
		T1	5.69 b	24.57 b	21.64 b	1.13 b
		T2	9.04 a	28.50 a	25.71 a	1.48 a
		T3	8.40 a	27.77 a	27.33 a	1.58 a
		T4	4.84 b	21.81 c	20.77 b	1.19 b
		Average	6.49	25.28	23.04	1.64
*p-value*		L	27.84**	6.75*	72.12**	16.01*
		T	10.25*	0.75*^ns^*	466.37**	10.01*
		L*T	2.46*^ns^*	0.37*^ns^*	3.78*^ns^*	11.09*

The content of total carbohydrates (starch, sucrose, and reducing sugars) were examined under two light levels in growing seasons of 2019 and 2020. T0, T1, T2, T3, and T4 denoted different ratios of NH_4_^+^/NO_3_^–^ (0/0, 0/100, 25/75, 50/50, and 75/25). Means in a column that do not share the same letters differ substantially at (LSD) (*p < 0.05). The results are mean ± SE (standard error). Values below ANOVAs are with a significance level of p ≤ 0.05, p ≤ 0.01 and ns, non-significant. The symbols “*” and “**” represent the significance level and the high significance level at a probability level of p ≤ 0.05 and p ≤ 0.01, respectively.

**FIGURE 5:**
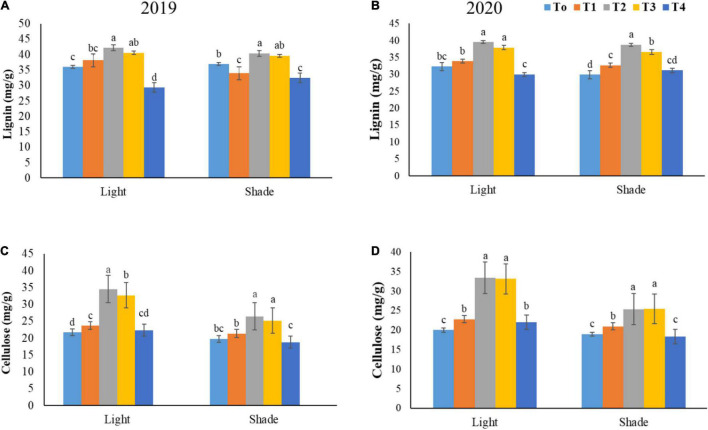
Effect of various NH_4_^+^/NO_3_^–^ ratios on lignin content **(A,B)**, and cellulose content **(C,D)** under light and shade. Different treatment levels T0, T1, T2, T3, and T4 characterize the ratios of NH_4_^+^/NO_3_^–^ 0/0, 0/100, 25/75, 50/50, and 75/25, respectively. All of the results are the mean of three replications. The bars represent the ± SD (standard deviation). A significant difference (*p < 0.05) between treatments is shown by different lowercase letters above bars.

The lignin and cellulose content were greatly influenced by the foliar application of NH_4_^+^/NO_3_^–^ ratios under shade conditions (Figure 5). As our results depicted that lignin content positively increased in both years (2019–2020) by 18.34 and 24.35% at T2 when compared to T0 and T4, respectively, under shade conditions. Our findings described that, lignin content significantly enhanced by 13.53 and 37.83% at T2 level as compared to T4 and T1 under normal light condition, respectively, for both years (2019–2020). In addition, a 62.89 and 57.71% increment in cellulose content was existed at T2 and T3 as compared to the untreated plants under normal light conditions. On the other hand, under shade conditions, the lignin content was reduced by 17.71 and 36.56% in T1 and T4 treatments in contrast to the T3 level, respectively, for both years. A 33.71% enhancement in cellulose content was exhibited by T2 treatment when compared with control plants under shade conditions in both growing seasons 2019–2020.

Similar to other studied parameters, the C/N ratio was also profoundly affected by different ratios of NH_4_^+^/NO_3_^–^ under shade and normal light regimes in both of the investigated years (2019–2020). Under a normal light environment, the C/N ratio was remarkably improved by 13.68% after T2 treatment when compared with T1 treatment in both years. Under shade conditions the C/N ratio was significantly enhanced by 21.22 and 15.10% at T2 and T3 treatments, observed relative to T0 treatment for both growing seasons (2019–20). Furthermore, in shading conditions, the T2 treatment induced a 5.31 and 20.35% increment in C/N ratio relative to T3 and T4, respectively, for both years (2019–2020).

### Lodging Resistance Index and Stem Snapping Resistance

**FIGURE 6:**
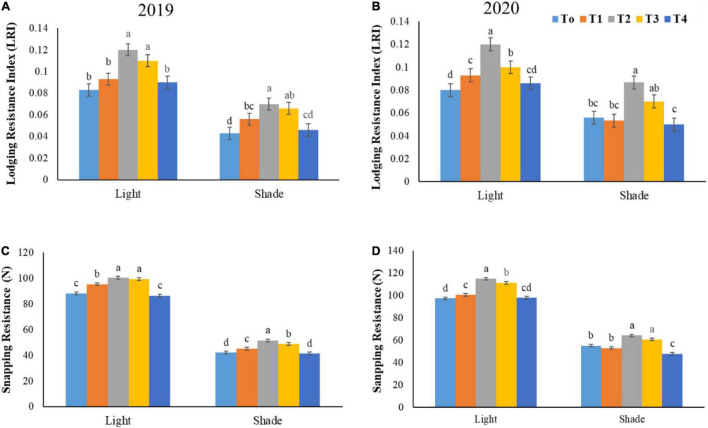
Effect of various NH_4_^+^/NO_3_^–^ ratios on lodging resistance index **(A,B)**, and snapping resistance **(C,D)** under light and shade. Different treatment levels T0, T1, T2, T3, and T4 characterize the ratios of NH_4_^+^/NO_3_^–^ 0/0, 0/100, 25/75, 50/50, and 75/25, respectively. All of the results are the mean of three replications. The bars represent the ± SD (standard deviation). A significant difference (*p < 0.05) between treatments is shown by different lowercase letters above bars.

A significant difference was found in snapping resistance and lodging resistance index between the plants grown in both light and shade conditions under different NH_4_^+^/NO_3_^–^ ratios (Figure 6). In both years 2019–2020, under normal light, the lodging resistance index accelerated in T2 by 29.03 and 36.36% as compared to T4 and T1, respectively. However, the upregulation of 9.84 and 7.49% in stem snapping resistance was seen in T2 and T3 treatment relative to T1, respectively. Under shade conditions, the higher acceleration in lodging resistance index and stem snapping resistance was observed under optimum NH_4_^+^/NO_3_^–^ ratios when compared to normal light regime. More specifically, under shade environment, the 44.44 and 59.18% improvement in lodging resistance index was exhibited by T2 treatment as compared to T0 and T1, respectively. Whereas, the T2 and T3 treatments improved the stem snapping resistance by 86.84 and 79.63% over T4 in both years 2019–2020.

### Chlorophyll Pigments and Relative Chlorophyll Content (SPAD) Value

Different NH_4_^+^/NO_3_^–^ ratios had significantly affected the chlorophyll pigments (chl a, chl b, carotenoids, and SPAD value) under both light regimes (Table 2). Our findings depicted that, under normal light conditions chl a, and chl b significantly increased by 32.02 and 50.56% at T2 treatment as compared to T4 in 2019. However, the SPAD value and carotenoids value showed increment by 10.74 and 95.65% at T2 as compared to T4 treatment in both years. Furthermore, under shade conditions chl a, carotenoids, and SPAD value were found to be increased by 15.58, 66, and 14.48% at T2 as compared to T1 treatment, respectively. While the chl b was improved at T3 by 36.75% as compared to T1 under shade conditions for both years (2019–2020). Interestingly, overall chlorophyll contents decreased with the increasing NH_4_^+^/NO_3_^–^ ratios under both light regimes (normal light and shade conditions).

**TABLE 2:** Effect varied NH_4_^+^/NO_3_^–^ ratios on the chlorophyll pigments under different light conditions.

Year	Treatments		Chl a	Chl b	Carotenoids	SPAD
2019	Normal light	T0	11.95 c	3.77 d	0.70 c	46.90 c
		T1	13.11 b	5.53 b	0.95 b	48.36 b
		T2	15.17 a	6.64 a	1.27 a	51.63 a
		T3	13.33 b	6.81 a	1.16 b	50.33 a
		T4	11.49 c	4.41 c	0.71 c	46.16 c
		Average	13.01	5.43	0.95	48.67
	Shade condition	T0	7.55 d	1.60 d	0.13 b	36.43 c
		T1	8.33 c	2.10 bc	0.18 b	37.53 bc
		T2	9.47 b	2.36 b	0.43 a	43.10 a
		T3	10.85 a	2.88 a	0.20 b	41.46 a
		T4	8.52 c	1.92 cd	0.21 b	39.30 b
		Average	8.94	2.17	0.23	39.56
2020	Normal light	T0	8.11 c	4.57 c	0.45 d	46.33 c
		T1	9.11 c	6.83 b	0.55 c	47.46 bc
		T2	13.23 a	8.59 a	0.98 a	52.60 a
		T3	11.50 b	9.07 a	0.87 b	51.53 a
		T4	7.37 c	5.67 bc	0.44 d	47.96 b
		Average	9.86	6.94	065	39.17
	Shade condition	T0	5.16 d	4.59 c	0.25 d	36.46 c
		T1	7.21 c	6.32 b	0.33 c	37.03 c
		T2	8.33 b	7.92 a	0.77 a	42.26 a
		T3	9.85 a	8.62 a	0.67 b	40.30 b
		T4	5.44 d	5.26 b	0.32 c	37.70 c
		Average	7.19	6.54	0.46	38.75
*p-value*		T	59.01**	50.53**	21.02**	34.8**
		L	87.6**	83.1**	45.1**	71.6**
		L*T	18.51**	12.91**	6.37*	0.838*^ns^*

Chlorophyll pigments and SPAD value were examined under two light levels in 2019–2020. T0, T1, T2, T3, and T4 denoted different ratios of NH_4_^+^/NO_3_^–^ (0/0, 0/100, 25/75, 50/50, and 75/25). Means in a column that do not share the same letters differ substantially at (LSD) (*p < 0.05). The results are mean ± SE (standard error). Values below ANOVAs are with a significance level of p ≤ 0.05, p ≤ 0.01 and ns, non-significant. The symbols “*” and “**” represent the significance level and the high significance level at a probability level of p ≤ 0.05 and p ≤ 0.01, respectively.

### Net Photosynthetic Rate

**FIGURE 7:**
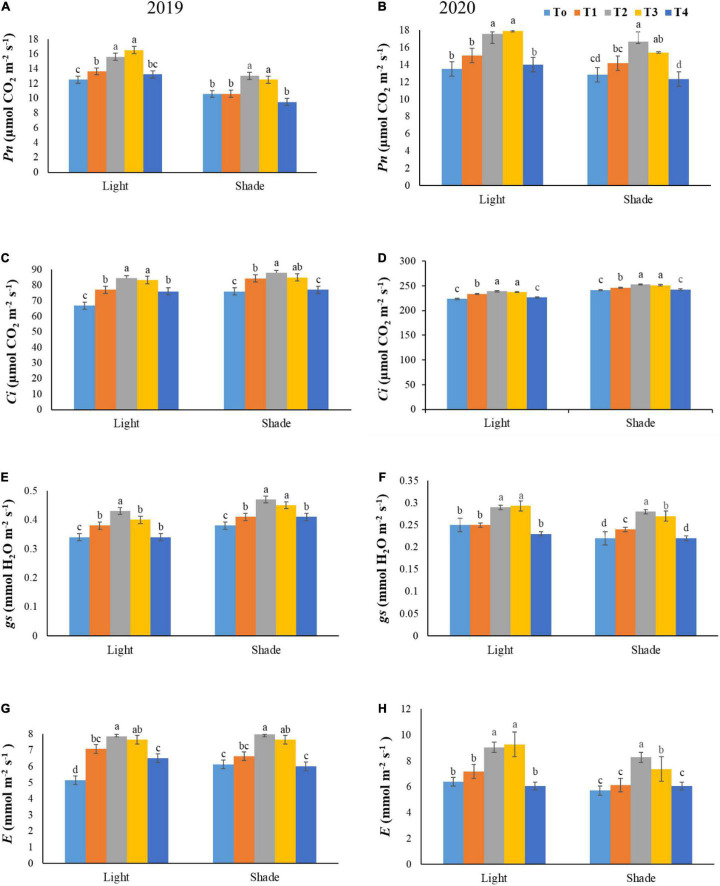
Effect of various ratios of NH_4_^+^/NO_3_^–^ on the net photosynthetic rate (*Pn*) **(A,B)**, intracellular CO_2_ (*Ci*) **(C,D)**, stomatal conductance (*gs*) **(E,F)**, and transpiration rate (*E*) **(G,H)** under both light and shade conditions. Different treatment levels T0, T1, T2, T3, and T4 characterize the ratios of NH_4_^+^/NO_3_^–^ 0/0, 0/100, 25/75, 50/50, and 75/25, respectively. All of the results are the mean of three replications. The bars represent the ± SD (standard deviation). A significant difference (*p < 0.05) between treatments is shown by different lowercase letters above bars.

Foliar application of NH_4_^+^/NO_3_^–^ remarkably influenced the *Pn*, *Ci*, *gs*, and *E* under both light environments. Under normal light the *Pn* and C*i* rate improved by 19.59 and 3.25% at T3 over T1, respectively. However, the 16.12 and 18.51% enhancement of values were shown by T2 treatment in contrast to the T1 in both years, respectively. Under shade treatment, the *Pn* and *E* rate enhanced by 35.93 and 35.26% at T2 as compared to T4 in both years. Moreover, the inhibition of C*i* and *gs* was observed with increasing NH_4_^+^/NO_3_^–^ ratios as it can be observed in Figure 7, the C*i* rate reduction at T4 and T1 by 3.10 and 6.59% as compared to T2, respectively. Under shading conditions, the *gs* rate significantly increased by 19.35% at T2 as compared to T4 level in both years 2019–2020.

### Photochemical Efficiency

**FIGURE 8:**
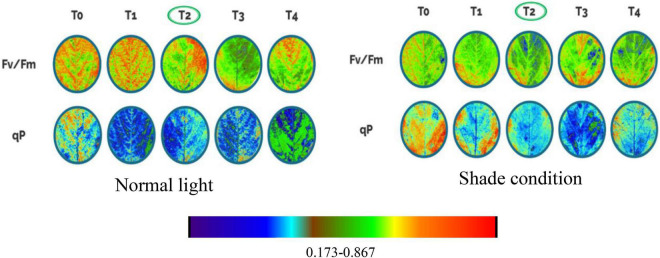
Impact of foliar application NH_4_^+^/NO_3_^–^ ratios on soybean photochemical efficiency under normal light and shade. Ratios of NH_4_^+^/NO_3_^–^ (T0, T1, T2, T3, and T4) denote 0/0, 0/100, 25/75, 50/50, and 75/25, respectively.

Shade stress negatively affected the photochemical efficiency including PS II (photosystem II), Fv/Fm (maximum quantum yield in dark), qP (photochemical quenching), and ETR (electron transport rate). Our findings demonstrated that optimum NH_4_^+^/NO_3_^–^ ratios (T2 and T3) significantly improved the photochemical activities of soybean plants under both light regimes (Table 3). Under the normal light condition, the PS II, Fv/Fm, qP, and ETR enhanced at T2 level by 23.80, 7.89, 6.81, and 12.55% over T4 treatment, respectively, in both years 2019–2020. While, under shade conditions, the values of PS II, Fv/Fm, qP, and ETR were increased at T2 when compared to T4 treatment by 11.36, 4.51, 8.43, and 7.39%, respectively, for both years (2019–2020) cumulatively (Figure 8). Additionally, the value of qP and ETR prompted by 7.55 and 9.75% at T2 as compared to T0 under shade conditions in both years 2019–2020.

**TABLE 3:** Effect of NH_4_^+^/NO_3_^–^ on the photochemical efficiency under various light conditions.

Year	Treatments (T)		Fv/Fm Ratio	PS II (mmol m^–2^ s^–1^)	qP (mmol m^–2^ s^–1^)	ETR (μ mol m^–2^s^–1^)
2019	Normal light	T0	0.74 b	0.22 c	0.45 b	100.41 cd
		T1	0.79 a	0.23 c	0.48 a	103.19 bc
		T2	0.81 a	0.26 a	0.48 a	106.09 a
		T3	0.79 a	0.24 b	0.47 ab	104.33 ab
		T4	0.74 ab	0.21 c	0.45 ab	98.70 d
		Average	0.77	0.23	0.46	102.54
	Shade condition	T0	0.77 bc	0.22 b	0.40 b	93.27 c
		T1	0.78 ab	0.21 b	0.41 b	96.58 bc
		T2	0.80 a	0.23 a	0.43 a	100.65 a
		T3	0.79 ab	0.22 ab	0.42 ab	98.16 ab
		T4	0.76 c	0.21 b	0.41 b	95.63 c
		Average	0.78	0.21	0.41	96.85
2020	Normal light	T0	0.813 bc	0.22 c	0.41 c	110.06 c
		T1	0.817 ab	0.24 b	0.437 bc	116.45 b
		T2	0.83 a	0.27 a	0.47 a	129.10 a
		T3	0.83 a	0.26 a	0.46 ab	125.95 a
		T4	0.79 c	0.224 bc	0.434 c	110.24 c
		Average	0.81	0.24	0.44	118.36
	Shade condition	T0	0.77 c	0.23 b	0.45 ab	101.86 b
		T1	0.79 bc	0.23 b	0.44 bc	102.86 b
		T2	0.82 a	0.26 a	0.47 a	109.22 a
		T3	0.81 ab	0.25 a	0.46 ab	108.59 a
		T4	0.76 c	0.23 b	0.42 c	101.58 b
		Average	0.79	0.24	0.44	104.82
*p-value*		T	0.7*	1.47*	0.71*	1.47*
		L	0.6*	9.35**	0.25**	9.35**
		L*T	0.7*	0.86*^ns^*	1.82*	0.86*^ns^*

Photochemical efficiency (PS II, Fv/Fm, qP, and ETR) values were examined under two light levels in 2019–2020. T0, T1, T2, T3, and T4 denoted different ratios of NH_4_^+^/NO_3_^–^ (0/0, 0/100, 25/75, 50/50, and 75/25). Means in a column that do not share the same letters differ substantially at (LSD) (*p < 0.05). The results are mean ± SE (standard error). Values below ANOVAs are with a significance level of p ≤ 0.05, p ≤ 0.01 and ns, non-significant. The symbols “*” and “**” represent the significance level and the high significance level at a probability level of p ≤ 0.05 and p ≤ 0.01, respectively.

### Lignin Related Enzymes Activities

The activity of lignin related enzymes was greatly influenced by various NH_4_^+^/NO_3_^–^ ratios under light and shade conditions in both years 2019–2020 (Table 4). Our results revealed that optimum NH_4_^+^/NO_3_^–^ ratios (T2 and T3) significantly enhanced the lignin enzymes activities under shade conditions and (T2 and T3) are recommended as the best ratios in comparison to higher NH_4_^+^/NO_3_^–^ ratios (T1 and T4). Under the light condition, the activity of POD, CAD, and 4Cl was positively up-regulated at T2 by 65.19, 13.73, and 28.72% as compared to T4 treatment, respectively. The minimum values for POD, CAD, and 4Cl were observed at T4 in 2019 (3.29 OD290/min/g), (0.92 nmol/min/g), (0.80 μ/g), and 2020 (4.81 OD290/min/g), (0.84 nmol/min/g), (1.04 μ/g), respectively. While, under shade condition, the 72.08, 8.13, and 10.60% increment was shown by the T2 treatment in contrast to the T1 for POD, CAD, and 4Cl, respectively.

**TABLE 4:** Effect of NH_4_^+^/NO_3_^–^ on the lignin related enzymes activity under different light conditions.

Year	Treatments (T)		POD (OD290/min/g)	CAD (nmol/min/g)	4Cl (μ /g)
2019	Normal light	T0	3.29 c	0.92 c	0.80 c
		T1	4.76 b	0.95 bc	0.93 b
		T2	7.02 a	1.06 a	1.20 a
		T3	6.25 a	0.99 a	1.13 a
		T4	3.37 bc	0.91 c	0.81 bc
		Average	4.93	0.96	0.97
	Shade condition	T0	3.60 b	0.81 b	0.41 c
		T1	3.86 b	0.85 b	0.46 b
		T2	6.29 a	0.94 a	0.51 a
		T3	5.60 a	0.93 a	0.49 a
		T4	3.97 b	0.72 c	0.40 c
		Average	4.66	0.85	0.45
2020	Normal light	T0	4.81 c	0.84 c	1.04 c
		T1	5.95 bc	0.87 b	1.09 bc
		T2	8.75 a	0.92 a	1.22 a
		T3	8.39 a	0.90 ab	1.16 bc
		T4	6.18 b	0.84 c	1.07 c
		Average	6.81	0.87	1.11
	Shade condition	T0	4.65 b	0.65 d	0.83 d
		T1	4.88 b	0.68 c	0.87 c
		T2	8.26 a	0.76 a	0.95 a
		T3	7.49 a	0.71 b	0.90 b
		T4	4.89 b	0.66 cd	0.81 d
		Average	6.03	0.69	0.87
*p-value*		T	27.0**	48.28**	29.66**
		L	2.01*	148.66**	74.06**
		L*T	1.37*^ns^*	1.44*	10.84**

Lignin related enzymes activity (POD, CAD, and 4Cl) values were examined under two light levels in 2019–2020. T0, T1, T2, T3, and T4 denoted different ratios of NH_4_^+^/NO_3_^–^ (0/0, 0/100, 25/75, 50/50, and 75/25). Means in a column that do not share the same letters differ substantially at (LSD) (*p < 0.05). The results are mean ± SE (standard error). Values below ANOVAs are with a significance level of p ≤ 0.05, p ≤ 0.01 and ns, non-significant. The symbols “*” and “**” represent the significance level and the high significance level at a probability level of p ≤ 0.05 and p ≤ 0.01, respectively.

### Yield Parameters

Likewise, different ratios of NH_4_^+^/NO_3_^–^ had a strong impact on yield parameters, including number of pods, 100 seed weight, and yield g/m^2^ (Table 5). Our investigations showed that optimum ratios (T2 and T3) enhanced the yield of soybean plants under both light and shade conditions in both the studied years. Under light conditions, the highest values for number of pods, 100 seed weight, and yield g/m^2^ were observed at T2 in 2019 (102.67, 16.13, and 264.53) and 2020 (105.33, 14.66, and 263.21), respectively. However, lowest number of pods, 100 seed weight, and yield g/m^2^ were recorded 85.00, 12.75, and 242.54 at T4 in 2020, and in 2019 the lowest values for 100 seed weight, and yield g/m^2^ were 12.70, and 244.87, respectively, while, the lowest number of pods (85.00) was observed at T0 treatment. Moreover, under shade conditions the maximum values for number of pods, 100 seed weight, and yield g/m^2^ were observed at T2 (92.33, 13.68, and 198.61) in 2019 and 2020 (75.00, 14.19, and 174.51), respectively. Conversely, the minimum values in 2019 for number of pods, 100 seed weight, and for yield g/m^2^ (70.33, 11.37, and 158.46) were found at T4, respectively, under normal light conditions. Additionally, the lowest values for number of pods and yield g/m^2^ 55.00 and 147.17 were shown by T4, respectively, and T0 experienced the lowest 100 seed weight (12.13) in the 2020 growing season under shade conditions.

**TABLE 5:** Effect of NH_4_^+^/NO_3_^–^ ratios on the yield parameters under different light conditions.

Year	Treatments (T)		No. of pods/plant	100 seed weight (g)	Yield (g/m^2^)
2019	Normal light	T0	85.00 c	13.06 bc	247.50 bc
		T1	93.33 b	14.22 b	246.37 c
		T2	102.67 a	16.13 a	264.53 a
		T3	102.33 b	15.63 a	252.18 b
		T4	86.33 c	12.70 c	244.87 c
		Average	93.93	14.34	251.09
	Shade condition	T0	77.66 b	11.72 b	160.62 b
		T1	80.33 b	12.13 b	151.60 c
		T2	92.33 a	13.68 a	198.61 a
		T3	89.66 a	13.09 a	196.65 a
		T4	70.33c	11.37 b	158.46 b
		Average	82.06	12.39	173.88
2020	Normal light	T0	84.00 d	12.75 b	248.76 c
		T1	87.00 c	13.18 ab	255.01 b
		T2	105.33 a	14.66 a	263.21 a
		T3	99.67b	13.85 ab	258.82 ab
		T4	85.00 cd	12.75 b	242.54 d
		Average	92.2	13.43	253.66
	Shade condition	T0	57.00 d	12.13 b	148.04 c
		T1	61.33 c	12.88 ab	149.77 c
		T2	75.00 a	14.19 a	174.51 a
		T3	70.00 b	13.54 ab	168.14 b
		T4	55.00 d	12.31 b	147.17 c
		Average	63.66	13.01	157.52
*p-value*		T	58.70**	19.36**	63.44**
		L	140.81**	79.78**	261.26**
		L*T	2.09*^ns^*	0.921*^ns^*	32.94**

Soybean yield characteristics including (number of pods per plant, 100 seed weight, and yield) were measured in response of varied NH_4_^+^/NO_3_^–^ ratios under light and shade conditions in both growing seasons 2019–2020. T0, T1, T2, T3, and T4 denoted different ratios of NH_4_^+^/NO_3_^–^ (0/0, 0/100, 25/75, 50/50, and 75/25). Means in a column that do not share the same letters differ substantially at (LSD) (*p < 0.05). The results are mean ± SE (standard error). Values below ANOVAs are with a significance level of p ≤ 0.05, p ≤ 0.01 and ns, non-significant. The symbols “*” and “**” represent the significance level and the high significance level at a probability level of p ≤ 0.05 and p ≤ 0.01, respectively.

### Correlation Analysis

**FIGURE 9:**
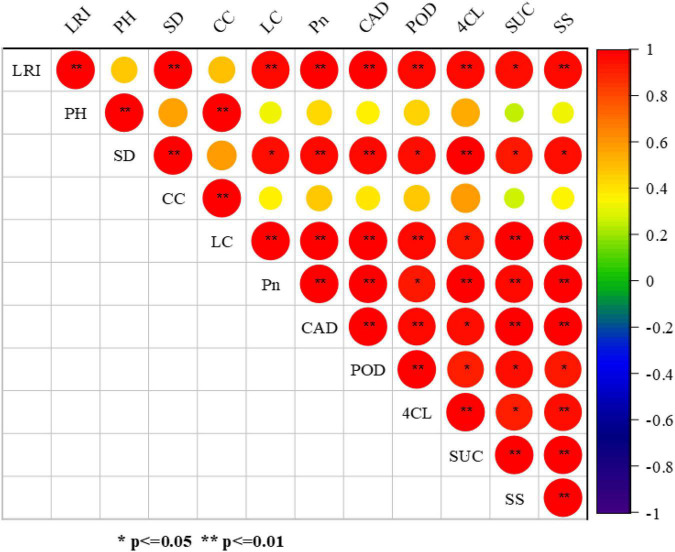
Relationship between stem characteristics, lignin content, and related enzyme activities. The following are the abbreviations used in the figure: lodging resistance index (LRI), and plant height (PH), stem diameter (SD), cellulose content (CC), lignin content (LC), photosynthetic rate (Pn), peroxidase enzyme activity (POD), cinnamyl alcohol dehydrogenase enzyme activity (CAD), 4-coumarate CoA ligase enzyme activity (4CL), sucrose content (SUC), and stem strength (SS) of soybean stem. The circle size showed the significant level and the circle color exhibited the positive or negative correlations. The significance difference among different parameters was showed by the *, ** at the probability level of *p* ≤ 0.05, *p* ≤ 0.01.

Correlation analysis depicted that lodging resistance index of soybean stem under shade conditions had a strong positive correlation with stem diameter, stem strength, lignin content, photosynthesis, and lignin related enzymes (POD, CAD, and 4CL) indicating the potential involvement of lignin and its related enzymes in the improvement of lodging resistance with a significance level of *p* ≤ 0.05, *p* ≤ 0.01 (Figure 9). However, the plant height and cellulose content did not show such a relationship. Furthermore, the significant positive correlation was observed with a significance level of *p* ≤ 0.05, *p* ≤ 0.01 between stem strength, sucrose content, photosynthesis, and lignin related enzymes (POD, CAD, and 4CL).

## Discussion

### Morphological Characteristics and Biomass Accumulation

A low light environment had drastic impacts on the soybean growth and development. The stem morphological characteristics including height, stem diameter, and stem snapping resistance were negatively affected under the shade stress (Raza et al., 2020). Our research depicted that the height of soybean plants increased, however, the stem diameter and snapping resistance decreased under shade stress. Our data clearly demonstrated that optimum ratios of NH_4_^+^/NO_3_^–^ (T2 and T3) significantly decreased the plant height and enhanced the stem diameter and snapping resistance. It was found that stem snapping resistance positively correlated with the lignin and cellulose content deposition in the base of stem (Hussain et al., 2019b). Our findings are consistent with those of Knapp et al. (1987), who found that higher N concentrations increased the lodging by increasing the inter-nodal base length, however, lower N levels increased stem diameter and helped with lodging resistance (Berry et al., 2000). Nonetheless, the phenomenon between the plant height and various nitrogen forms and ratios under shade condition is less studied. The height of plant decreased under optimum ratios of NH_4_^+^/NO_3_^–^ (T2 and T3) and with increasing the concentration of nitrogen, the height decreased below the optimum level. This might be due to the auxin and gibberellic acid production at the stem tips, thus it requires further deeper molecular studies about the relationship between the auxin and gibberellic acid metabolism under shade stress due to shade avoidance response of plants (Bawa et al., 2020; Gho et al., 2021). Furthermore, our findings are in-line with previous study that stem snapping resistance related with stem diameter and stem weight (Hussain et al., 2021), since the increment of stem diameter and snapping resistance was also observed in our study by the ratios of NH_4_^+^/NO_3_^–^ (T2 and T3).

Shading is the most significant chronic constraint in maize-soybean intercropping, with long-term consequences for soybean growth and development (Yao et al., 2017b). Our studies evaluated that varied NH_4_^+^/NO_3_^–^ ratios affected the soybean growth and physiochemical activities. The results of our study exposed that optimum NH_4_^+^/NO_3_^–^ (T2 and T3) ratios greatly improved the fresh biomass production of soybean seedlings, which was also reported in our recent findings under various light circumstances (Raza et al., 2021). This might be because low light conditions result in less carbohydrate buildup due to reduced carbon metabolism which required less ammonium concentration to boost biomass synthesis (Hu et al., 2017). Tabatabaei et al. (2008) also found that optimum NH_4_^+^/NO_3_^–^ ratios improved the plant growth in both normal and low light situations. In addition, Chen et al. (2013) found that an optimal mixture of NH_4_^+^/NO_3_^–^ increased plant growth and biomass output substantially. In our study, plants with greater NH_4_^+^/NO_3_^–^ ratios (T1 and T4) had a detrimental influence on dry biomass production under both light regimes. This might be because under low light, NH_4_^+^ accumulated in the leaves which resulted in the uncoupling of electron transport from the process of photophosphorylation in the chloroplasts, and as consequence, photosynthetic rate decreased which ultimately led to declined biomass accumulation (Hu et al., 2015). However, Hu et al. (2017) also described that under shade conditions low light intensity reduced the carbon metabolism which resulted in less carbohydrate accumulation in plant leaves.

### Response of Photosynthetic and Photochemical Activities and Chlorophyll Pigments to Different NH_4_^+^/NO_3_^–^ Ratios

Nitrogen can significantly prompt the capability of photosynthetic processes comprising *Pn*, gs, *E*, and C*i* (Huang et al., 2011; Feng et al., 2012). The present study demonstrated that foliar sprays of NH_4_^+^/NO_3_^–^ with optimum levels (T2 and T3) improved the photosynthetic activities compared to higher levels of NH_4_^+^/NO_3_^–^ (T1 and T4) under shade conditions. It has been reported previously that the shaded plants fed with higher NH_4_^+^ ratios decreased the photosynthetic rate which is due to ammonium toxicity in the leaves (Esteban et al., 2016). Furthermore, it has been previously reported that plants treated with NH_4_^+^ solely exhibited increased photosynthetic activity in low light conditions due to enhanced protein production (Golvano et al., 1982). However, in our study a reduction in stomatal conductance (*g*_*s*_) was observed under shade conditions which caused inhibition in intercellular CO_2_ (*C*_*i*_) which is in agreement with a previously reported study (Cruz et al., 2013). Another study demonstrated that, in nutrient solutions containing high levels of NH_4_^+^/NO_3_^–^, the stomatal conductance (*gs*) decreased owing to an imbalanced absorption of ions caused by the absence of cation (NH_4_^+^) or anion (NO_3_^–^) that reduced photosynthetic activities (Lopes and Araus, 2006). Therefore, for maximum biomass production nitrogen ratios and light intensity should be appropriate. Our findings reported that optimum NH_4_^+^/NO_3_^–^ ratios (T2 and T3) improved the photosynthetic activities which resulted in maximum carbohydrates and biomass production and ultimately enhanced the lodging resistance of soybean stem under shade conditions.

Because of its sensitive reaction on plants and the environment, chlorophyll fluorescence has to be the most significant indicator for evaluating the performance of photosynthetic processes (Dai et al., 2009). Our findings showed that optimum NH_4_^+^/NO_3_^–^ (T2 and T3) ratios significantly improved the performance of fluorescence metrics such as PSII, F/Fm, qP, and ETR for both light conditions as depicted in Figure 8. In our study, under shade conditions plants sprayed with higher NH_4_^+^/NO_3_^–^ (T1 and T4) might have a reduced light capture area due to chloroplast damage which is consistent with another study (Hachiya et al., 2012). On the other hand, plants treated with optimum NH_4_^+^/NO_3_^–^ (T2 and T3) ratios under shade conditions significantly enhanced the photochemical efficiency of photosystem II (PSII) by up-regulating the carboxylation rate and ATP generation rate (Hu et al., 2020). Previous research has shown that shade reduces photosynthesis and, as a result, decreases the chlorophyll content of plants (Yao et al., 2017a). Chlorophyll also exhibited a significant function in indicating plant development and photosynthesis rate in response to various nitrogen ratios and concentrations. Plants treated with high levels of NH_4_^+^/NO_3_^–^ (T1 and T4) had a detrimental influence on chlorophyll and total carbohydrate contents under both light conditions. Consistent with our results, a study revealed that high ratios of NH_4_^+^/NO_3_^–^ raptured the thylakoid membranes and photosystem II, affecting energy transfer from antennas to photochemical reactions and lowering the Fv/Fm rate (Khamis et al., 1990). The outcomes of our study also corroborated the findings of Hu et al. (2017), which showed that optimum ratios of NH_4_^+^/NO_3_^–^ significantly boosted the process of carboxylation and photochemical quenching. Collectively, our study depicted that optimum NH_4_^+^/NO_3_^–^ (T2 and T3) ratios can inhibit the negative effects of shade on the photochemical activities and resulted in improved photosynthesis process and biochemical activities which ultimately enhanced the lodging resistance of soybean stem under shade conditions.

### Lignin Enzymes, Stem Strength, and Lodging Resistance Index

This research portrayed that, the lignin and cellulose content significantly reduced at basal stem under shade stress as reported previously (Hussain et al., 2019a). Our results showed that the content of lignin and cellulose was significantly positive correlated with lodging resistance index. The findings of our study indicated that optimum (T2 and T3) or reduced nitrogen fertilization increase the lignin and cellulose content, and enhanced the lodging resistance index. Similarly, the previous studies also suggested that the enhancement of lodging resistance index was mainly due to increasing the content of lignin and cellulose (Liu et al., 2021). Moreover, our findings also demonstrated that higher concentrations of nitrogen fertilizers reduced the lignin and cellulose content which resulted in the higher risks of lodging stress for soybean plants (Zhang et al., 2017a). Lodging is a dynamic attribute that is linked to a variety of plant traits. Such as, yield composition also significantly correlated to the lodging resistance, as lodging resistance accelerates the yield, which was also improved. It is understandable that stems which are strong could actively transport the photosynthetic assimilates and nutrients and result in a higher grain yield (Muhammad et al., 2020). The relationship between lodging resistance of soybean stem and different NH_4_^+^/NO_3_^–^ ratios and its mechanisms were studied on the basis of morpho-physiological and biochemical activities. As depicted in our research, the efficacy of photosynthetic apparatus was increased in response to optimum NH_4_^+^/NO_3_^–^ ratios (T2 and T3) as compared to higher ratios (T1 and T4), which resulted in maximum production of lignin and cellulose content under shade conditions. Similarly, previous studies described that higher application of nitrogen reduced the non-structural carbohydrates content in the stems and also decreased the lignin content which is an agreement with our findings (Kuai et al., 2016; Zhang et al., 2017b).

**FIGURE 10:**
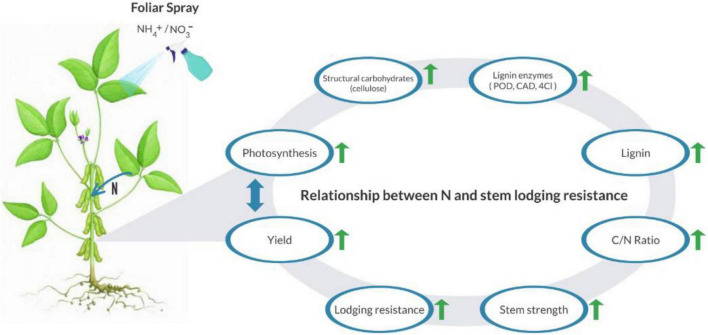
An illustration exhibiting that, different NH_4_^+^/NO_3_^–^ ratios up-regulated the physiological and biochemical mechanisms and ultimately enhanced the lodging resistance of soybean stem and yield. The upward green arrow shows the up-regulation of the following parameters and double head blue color arrow depicts the relationship between photosynthesis and yield.

Shade stress significantly affected the activity of lignin enzymes (POD, CAD, and 4Cl) (Wen et al., 2020). Our findings revealed that higher ratios of NH_4_^+^/NO_3_^–^ decreased the lignin enzymes activities which is consistent with the findings of Wang et al. (2015), who reported that higher nitrogen fertilizers reduced the activities of enzymes such as PAL, POD, and 4CL. In addition, our study portrayed that optimum NH_4_^+^/NO_3_^–^ ratios (T2 and T3) up-regulated lignin related enzymes activities including PAL, POD, and 4CL which resulted in enhanced lignin content in soybean stem. However, under higher NH_4_^+^/NO_3_^–^ ratios (T1 and T4) the lignin enzymes activities were down-regulated which were confirmed by various studies (Moura et al., 2010; Wen et al., 2020). It was reported that higher concentration of NH_4_^+^/NO_3_^–^ reduced the lignin content due to β–O–4 linkage reduction and maximum ρ-hydroxyphenyl frequency units (Pitre et al., 2007; Moura et al., 2010; Luo et al., 2019). Our study exposed that, optimum NH_4_^+^/NO_3_^–^ ratios enhanced the lodging resistance of soybean stem by regulating enzymatic activities (PAL, POD, and 4CL) and physiological processes (photosynthesis and photochemical) under shade conditions. To portrayed the effects of NH_4_^+^/NO_3_^–^ ratios on the lodging resistance of soybean stems a proposed mechanism illustrated in Figure 10.

### Total Carbohydrates Content and Yield

The use of appropriate fertilizer ratios can improve the nitrate remobilization efficiency and thus maximize the N utilization for plant growth and grain yield. A recently reported study also proved that maximum yield could be attained using a low nitrogen fertilization through nitrate remobilization (Dong and Lin, 2020). Our research exhibited that, under shade conditions optimum NH_4_^+^/NO_3_^–^ ratios (T2 and T3) positively enhanced the carbohydrates content as compared to higher ratios (T1 and T4). It might be due to that, optimum ratios (T2 and T3) improved the photosynthetic process under shade conditions compared to higher ratios. As, higher ratios (T1 and T4) caused NH_4_^+^ toxicity in leaves and rapture the thylakoid membranes which inhibit the photochemical and photosynthetic efficiency and resulting in low carbohydrates and yield (Hu et al., 2015). Our findings showed that optimum ratios (T2 and T3) of foliar nitrogen application improved the soybean yield significantly. In this study, optimum ratios of NH_4_^+^/NO_3_^–^ (T2 and T3) raised the number of pods, 100 grain weight and ultimately the yield of soybean. It might be because of the higher starch content, maximum photosynthetic activities, and increased lodging resistance index due to higher lignin and cellulose content (Gao et al., 2017). The physiological parameters indicate that carbohydrate and nitrogen transport to the grain sink and they can also be quantified using spectroscopic methods (Liu et al., 2017a). These outcomes will help in the understanding of impact of different nitrogen forms (NH_4_^+^/NO_3_^–^) on lodging resistance of soybean plants under shade conditions, allowing new genetic strategies to be developed to alter stem architecture and eliminate the fundamental cause of excessive N demand in modern semi-dwarfing types, thus increasing production and reducing environmental pollution and economic risks.

## Conclusion

Conclusively, a significant (*p* ≤ 0.05, *p* ≤ 0.01) correlation was seen between stem strength, carbohydrate content, photosynthesis, and lignin related enzymes (POD, CAD, and 4CL). The growth of soybean including dry biomass accumulation, chlorophyll content, photosynthetic rate, and carbohydrates content were affected by higher NH_4_^+^/NO_3_^–^ ratios, leading to the stunted growth of soybean plants. However, the optimum ratios of NH_4_^+^/NO_3_^–^ (T2 and T3) increased the photosynthetic rate (19.88 and 12.36%), dry mass accumulation (23.38 and 17.45%), lignin content (18.66 and 14.31%) under shade condition when compared to higher ratio (T1) in 2019–2020 cumulatively. Similarly, the lodging resistance index was enhanced at the optimum NH_4_^+^/NO_3_^–^ ratios (T2 and T3) by an average of 62.5 and 41.66% as compared to higher ratio (T4) under shade conditions cumulatively in both years 2019–2020. Conversely, the optimum NH_4_^+^/NO_3_^–^ ratio (T2) also remarkably improved the lodging resistance by accelerating the lignin related enzymes activities (POD, CAD, and 4Cl). The soybean yield was observed at a maximum (263.87 and 186.56 g/m^2^) at optimum NH_4_^+^/NO_3_^–^ ratio (T2) under normal light and shade conditions, respectively, an average of both years (2019–2020). Therefore, our findings suggest that the optimum treatments (T2 and T3) related ratios of NH_4_^+^/NO_3_^–^ are recommended to improve the lodging resistance of soybean under shading conditions which is strong evidence for sustainable agriculture.

## Data Availability Statement

The original contributions presented in this study are included in the article/supplementary material, further inquiries can be directed to the corresponding author.

## Author Contributions

AR, WL, BC, MA, MI, and CY: roles, writing – original draft, review and editing, and data analysis. IS, HJ, MI, NK, TI, and AG: data curation and plotting. WL and WY: funding acquisition. All authors read the manuscript and approved the submitted version.

## Conflict of Interest

BC was employed by Chengdu Da Mei Seeds Co., Ltd. The remaining authors declare that the research was conducted in the absence of any commercial or financial relationships that could be construed as a potential conflict of interest.

## Publisher’s Note

All claims expressed in this article are solely those of the authors and do not necessarily represent those of their affiliated organizations, or those of the publisher, the editors and the reviewers. Any product that may be evaluated in this article, or claim that may be made by its manufacturer, is not guaranteed or endorsed by the publisher.
